# BOne HEalth ManagEment in Patients with Early Breast Cancer: A Retrospective Italian Osteoncology Center “Real-Life” Experience (BOHEME Study)

**DOI:** 10.3390/jcm8111894

**Published:** 2019-11-06

**Authors:** Federica Recine, Alberto Bongiovanni, Flavia Foca, Laura Mercatali, Valentina Fausti, Sebastiano Calpona, Nada Riva, Alessandro De Vita, Chiara Liverani, Chiara Spadazzi, Giacomo Miserocchi, Giandomenico Di Menna, Lorena Gurrieri, Claudia Cocchi, Silvia Angela Debonis, Roberto Vespignani, Toni Ibrahim

**Affiliations:** 1 Osteoncology and Rare Tumors Center, Istituto Scientifico Romagnolo per lo Studio e la Cura dei Tumori (IRST) IRCCS, 47014 Meldola, Italy; alberto.bongiovanni@irst.emr.it (A.B.); laura.mercatali@irst.emr.it (L.M.); valentina.fausti@irst.emr.it (V.F.); sebastiano.calpona@irst.emr.it (S.C.); nada.riva@irst.emr.it (N.R.); alessandro.devita@irst.emr.it (A.D.V.); chiara.liverani@irst.emr.it (C.L.); chiara.spadazzi@irst.emr.it (C.S.); giacomo.miserocchi@irst.emr.it (G.M.); giandomenico.dimenna@irst.emr.it (G.D.M.); lorena.gurrieri@irst.emr.it (L.G.); claudia.cocchi@irst.emr.it (C.C.); silviaangela.debonis@irst.emr.it (S.A.D.); toni.ibrahim@irst.emr.it (T.I.); 2 Unit of Biostatistics and Clinical Trials, Istituto Scientifico Romagnolo per lo Studio e la Cura dei Tumori (IRST) IRCCS, 47014 Meldola, Italy; flavia.foca@irst.emr.it; 3 IT Unit, Istituto Scientifico Romagnolo per lo Studio e la Cura dei Tumori (IRST) IRCCS, 47014 Meldola, Italy; roberto.vespignani@irst.emr.it

**Keywords:** bone health, breast cancer, hormone therapy, bone-modifying agents, denosumab, zoledronic acid

## Abstract

Background: We assessed the real-life clinical impact of bone health management in patients with breast cancer (BC) receiving adjuvant endocrine therapy at an Italian Osteoncology Center. Methods: Pre- and post-menopausal women undergoing adjuvant endocrine therapy for early-stage BC who came to our institute for their first bone health evaluation from January 2011 to June 2016 were considered in this retrospective observational study. Results: 1125 pre- and post-menopausal early-stage BC patients (209 and 916, respectively) were evaluated. Median age was 61 years (range 26–88). In the pre-menopausal group, spinal x-ray revealed that 10 patients (4.7%) had a morphometric vertebral fracture. Higher age (OR: 1.14; 95% CI: 1.01–1.29) and bone mineral density (BMD) ≤ −2.5 (OR: 14.45; 95% CI: 1.70–122.67) were associated with a higher risk of bone fracture. The overall frequency of bone fracture was 17.6% (n = 161) in post-menopausal patients and a lower risk for bone fractures was associated with tamoxifen or other treatments (OR: 0.25; 95% CI: 0.12–0.53), presence of back pain (OR: 1.65; 95% CI: 1.16–2.36), lower BMD (OR: 2.09 in patients with T-score ≤ 2.5; 95% CI: 1.21–3.59) and lower vitamin D levels (OR: 1.57 in patients with ≤ 10 ng/mL; 95% CI: 1.05–2.34) in univariate analysis. Conclusion: Our findings confirm that bone health management should be an integral part of long-term cancer care.

## 1. Introduction

Breast cancer (BC) is the most frequently diagnosed tumor in women worldwide whose survival is increasing thanks to improvements in treatment outcomes [1]. Around 80% of BCs express hormone receptors and can thus benefit from hormone treatments including aromatase inhibitors (AIs), selective estrogen receptor modulators (SERMs), such as tamoxifen (TAM), and from the surgical suppression of ovarian function by oophorectomy or treatment with luteinizing hormone-releasing hormone (LHRH) agonists [2,3,4]. Endocrine therapy (ET) creates an estrogen-deficient environment that induces changes in bone metabolism and alterations in bone homeostasis, leading to a loss of bone mass [5]. BC patients show an increased risk of bone fractures which most commonly occur in lower limbs and vertebral sites [6,7,8]. 

In this scenario, the evaluation of bone health is crucial to the optimal management of early-stage BC in preventing cancer treatment-induced bone loss (CTIBL). This phenomenon impairs the balance of bone tissue microarchitecture, leading to a systemic skeletal disorder characterized by reduced bone mineral density (BMD), high bone turnover, and increased bone fragility, all of which increase the risk of fractures occurring without significant trauma or even in the absence of trauma. CTIBL is associated with substantial financial costs and a high disease burden that negatively affects quality of life and increases patient mortality [9,10].

Current clinical recommendations underline the importance of baseline bone health evaluation in patients with early-stage BC to reduce the risk of bone fractures and improve clinical outcome. The assessment of the risk of bone fractures and bone loss comprises an appraisal of clinical risk factors for fractures and BMD measurements [11,12,13,14,15]. 

Bone loss can be treated or prevented with bone-modifying agents (BMAs) such as including bisphosphonates and denosumab which use different mechanisms to inhibit osteoclast activity and bone resorption, thereby increasing bone mineral density and reducing the likelihood of bone fracture. Moreover, these agents have been shown to improve clinical outcome [16,17,18,19].

Studies on preclinical models have revealed that BMAs may also impact the development of bone metastases through crosstalk between tumor and host cells within the bone marrow. This mechanism seems to have a role in the survival of tumor cells within the bone marrow, suggesting that these agents also have an impact on clinical outcome [20,21,22].

To our knowledge only a handful of small studies have focused on bone health in BC, looking at the correlation between bone fractures and primary hormone therapy in conjunction with prior bone morbidity and life style factors in patients with early-stage disease [8,23]. In this retrospective monocenter observational study, we aimed to evaluate the real-life clinical impact of bone health management in terms of morphometric bone fractures and clinical outcome in early BC patients undergoing endocrine therapy at an Italian Osteoncology Center. 

## 2. Patients and Methods

We identified pre-menopausal and post-menopausal women treated with adjuvant endocrine therapy for early-stage hormone receptor-positive BC (stages I, II, and III) at Istituto Scientifico Romagnolo per lo Studio e la Cura dei Tumori (IRST) IRCCS who came to the Osteoncology Clinic for their first bone health evaluation between January 2011 and June 2016. Patients with bone metastases and those without x-rays were excluded from the analysis. The evaluation consisted in the identification of risk factors for bone fractures such as previous ET, bone mineral density (BMD), and comorbidities. A blood sample was also taken to assess calcium metabolism, parathyroid hormone (PTH) and 25-hydroxyvitamin D levels, and carboxy-terminal telopeptides of type I collagen (CTX). All patients underwent a general physical examination and the body mass index (BMI) was calculated. 

Patients were asked to complete a self-reporting questionnaire during the visit to collect information on life style factors such as dietary patterns (milk, cheese, yoghurt, vegetables, and water), physical activity (established as at least 30 min/day), presence of back pain, BMD, and previous/current calcium therapy and ET. Information on smoking and alcohol habits was collected on the basis of the following categories: Current or past smoker and never smoker; no alcohol intake; half a glass of wine during a meal, corresponding to 0.5 g alcohol; and consumption of more than 0.5 g during a meal. For the analysis, patients with no alcohol consumption versus 0.5 g alcohol consumption during a meal were considered.

A short questionnaire on calcium intake was administered [24]. An adequate daily dietary calcium intake corresponding to the recommended daily allowance (RDA) of calcium was defined as two or more servings/day of milk, cheese or yoghurt, or at least one liter of water/day. The presence of morphometric vertebral deformities was assessed by spinal or lateral chest x-ray according to the Genant classification. BMD was determined by dual-energy x-ray absorptiometry (DXA) scan performed elsewhere or by quantitative ultrasound (QUS) carried out during the physical examination. In accordance with World Health Organization (WHO) criteria, the T-score was calculated as the number of standard deviations (SDs) above or below the average value for young healthy women. A T-score of ≤ −2.5 SDs was defined as osteoporosis, while a T-score between −1 and −2.5 was defined as osteopenia. Normal BMD was a T-score of ≥ −1.0. Concomitant medications and laboratory test results (serum levels of vitamin D, PTH, and CTX) were evaluated and recorded. Intact PTH values assessed with an electrochemiluminescence immunoassay, values between 11 and 67 ng/mL being considered normal. 

Clinical and radiological follow-up appointments were scheduled every two years thereafter to monitor bone health status and adherence to treatment with bone-modifying agents. A specific follow up regimen was recommended including laboratory exams (serum levels of calcium, vitamin D, PTH, CTX) every 12 months and BMD evaluation with DXA scan or QUS with spinal or chest X-ray every 12–18 months. All the information collected during the bone health visit was recorded in an electronic database, as were the results from an anonymous questionnaire on patient satisfaction completed at the end of the visit.

The study was approved by the Institutional Review Board of Istituto Scientifico Romagnolo per lo Studio e la Cura dei Tumori (IRST) IRCCS (protocol no. 174.20) and was conducted in accordance with the ethical standards laid down in the 1975 Declaration of Helsinki. The need for written informed consent from participants was waived because of the retrospective nature of the research. 

### Statistical Analysis

Separate analyses were carried out for pre-menopausal and post-menopausal women. Categorical variables were expressed as frequencies and percentages, while continuous variables were presented using median and range. Univariate and multivariate logistic models were used to obtain the odds ratios (ORs) for morphometric fractures and 95% confidence intervals (95% CI) were calculated. Independent variables that proved significant in univariate analysis were selected for inclusion in the multivariate model. A secondary explorative objective of distant relapse-free survival (DRFS) was calculated for a subgroup of patients with a follow-up of at least five years, defined as the time from the date of surgery to the date of distant relapse, or death from any cause. Event-free patients were censored on the date of the last follow-up. Survival curves were estimated using the product-limit method of Kaplan–Meier method (two-sided 95% CIs). Two-tailed p-values < 0.05 were considered statistically significant.

No corrections for multiple testing were performed. Explorative analyses on the role of CTX were carried out. Receiver operating characteristic (ROC) curves were generated and an area under the ROC curve (AUC) was plotted to determine the ability of CTX value to discriminate between subjects with or without bone fracture and with or without distant relapse. Statistical analyses were carried out with Stata/MP 10.1 for Windows (StataCorp LP, College Station, TX, USA).

## 3. Results

A total of 1125 pre- and post-menopausal women with early-stage BC treated with endocrine therapy were identified. Two-hundred and six patients were excluded due to a lack of x-rays and one because of bone metastasis. Patient characteristics are summarized in Table 1. Median age was 61 years (range 26–88 years). Two hundred and nine patients were pre-menopausal and 916 were post-menopausal. 

The information collected on daily dietary calcium intake showed that 67.0% of pre-menopausal patients and 74.6% of the post-menopausal group had a daily calcium intake less than that of the RDA. A similar proportion of women in both groups engaged in regular physical activity (67.5% and 65.2%, respectively).

Overall, 707 (62.8%) patients had undergone a DXA scan to evaluate BMD elsewhere, usually no more than six months before the bone health visit, while 339 (37.2%) patients underwent QUS at our Center during the visit. Data on univariate and multivariate analyses of the risk of bone fracture in pre-menopausal women are shown in Table 2. Around 4.7% of women had a morphometric vertebral fracture shown at x-ray of the spine or lateral. Higher age (OR: 1.14; 95% CI: 1.01–1.29) and BMD ≤ −2.5 (OR: 14.45; 95% CI: 1.70–122.67) were associated with a higher risk of bone fracture. Data from multivariate analysis confirmed that a lower BMD was an independent risk factor for bone fracture, with an OR of 11.6 (95% CI: 1.34–101.1) for patients with osteoporosis. 

The results of univariate and multivariate analyses for the risk of morphometric vertebral deformities in post-menopausal women are shown in Table 3. 17.6% of women had a morphometric vertebral fracture detected by spinal or lateral chest X-ray. The risk of morphometric vertebral deformities in post-menopausal women was correlated with higher age (OR: 2.53; 95% CI: 1.78–3.60), and BMD ≤ −2.5 (OR: 2.09; 95% CI: 1.21–3.59). 

Patients who engaged in regular physical activity had a 35% reduced risk of bone fracture (OR: 0.65; 95% CI: 0.46–0.92), while those with a BMI between 25 and 29 showed a greater risk (OR: 1.82; 95% CI: 1.23–2.67). A similar situation was observed for patients with BMI ≥ 30 (OR: 1.77; 95% CI: 1.09–2.86). Low levels of 25-hydroxyvitamin D were associated with a higher risk of bone fracture (OR: 1.57; 95% CI: 1.05–2.34) and the presence of back pain (OR: 1.65; 95% CI: 1.16–2.36). 

Our results showed that TAM had a protective effect (OR: 0.25; 95% CI: 0.12–0.53) against bone fractures compared to AI treatment. One hundred and forty-four post-menopausal patients were already undergoing treatment with BMAs due to a pre-existing condition of osteoporosis or the presence of pre-ET high-risk factors for morphometric vertebral deformities, including prolonged corticosteroid therapy (> 3 months) and previous bone fragility fractures. Patients who had taken BMAs for longer (n = 139) had a lower BMD (57.5%) than those with osteopenia (T score between −1.0 and −2.5) (41.1%). A small number of patients (1.4%) with normal BMD had undergone previous treatment with BMAs for other reasons, including pre-existing bone fractures and prolonged corticosteroid therapy; patients without previous treatment with BMAs (n = 694) had mostly a T score between −1.0 and −2.5) (49.4%), respect to patients with osteoporosis (29.7%) and patients with normal BMD (20.9%): the association between BMA treatment and T-score was significant (*p* ≤ 0.001).In multivariate analysis of post-menopausal patients, prior treatment with BMAs was not included because of the correlation between BMA therapy and lower BMD. In the post-multivariate analysis for menopausal group, higher age, the presence of back pain, and BMD ≤ −2.5 remained independent risk factors for bone fractures, whereas TAM showed a protective effect.

The median follow-up for the overall series was 61 months (range 7–403 months). DRFS was evaluable in 778 (69%) patients with a follow-up of ≥ 5 years, and 52 cases of distant recurrence were identified five years after diagnosis in the overall population. Five-year DRFS was 95.7% (95% CI: 90.8–98.1) for pre-menopausal women and 96.1% (95% CI: 94.2–97.5) for post-menopausal cases. 

Among 631 post-menopausal patients with at least five years of follow up, 44 progression of disease were observed. About 12.5% of patients with bone fractures during adjuvant ET, showed progressive disease (PD) compared to the 5.6% of women who did not have skeletal fractures. One, year DRFS was 99.8% (95% CI: 98.9–99.9) while three and five was 99.2% (95% CI: 98.0–99.7) and 96.1 (96% CI: 94.2–97.5) for post-menopausal women.

Exploratory analysis of CTX values revealed that they were not predictive of the risk of bone fractures (AUC: 0.59, 95% CI: 0.33–0.85 for pre-menopausal women, and AUC: 0.50, 95% CI: 0.44–0.57 for post-menopausal women) or distant disease recurrence (AUC: 0.68, 95% CI: 0.53–0.82 for pre-menopausal women, and AUC: 0.53, 95% CI: 0.42–0.63 for post-menopausal women) in this BC cancer population.

The anonymous data collected on patient satisfaction (1105 patients completed the questionnaire) revealed that 91.9% were highly satisfied with the osteoncology visit and 7.5% were fairly satisfied. Only 0.5% expressed low satisfaction. 97.7% of patients thought that the clinical bone health evaluation was very useful, 1.8% not very useful and 0.5% not useful at all.

## 4. Discussion

Bone homeostasis is a dynamic process that represents a balance between the activity of osteoblasts, which form bone, and osteoclasts, which resorb it. Alterations in bone homeostasis induced by cancer treatments, including ET, can lead to CTIBL, resulting in a break of normal skeletal structural integrity associated with increased bone turnover and a higher risk of skeletal fractures. The process of increased bone turnover is involved in both tumor growth and clonal expansion, with cellular interactions between tumor cells and other cell types found in the bone microenvironment, similar to the process that occurs during bone metastasization [20,21,22,25]. The “seed and soil” theory of metastasis suggests that cancer cells from the primary tumor the inherent potential to seek conditions similar to their original environment in which to grow and create new bone lesions [26,27].

In the adjuvant setting, the use of BMAs has the main aim of inhibiting bone loss and preventing adverse effects of cancer treatments on bone health. These agents have been shown to modify the underlying disease, blocking the development of metastases, and improving clinical outcome [16,17,18,19,28].

In the present retrospective study, we investigated the clinical impact of bone health management on a large population of pre- and post-menopausal early BC patients treated with ET, evaluated by a dedicated oncologist during their first visit to our institute’s Osteoncology Center. As expected, older age and lower BMD were correlated with a higher risk of bone fractures, as was ET with AIs. Other clinical factors correlated with an increased risk of bone fractures included low levels of 25-hydroxyvitamin D, back pain, and lack of physical activity. 

Our findings brought to light an unexpected relationship between BMI and risk of bone fracture. As reported elsewhere, the correlation between BMI and bone fracture is somewhat controversial [29,30]. The direct effect of BMI on a female population with BC and a risk of CTIBL would seem to be more complex than that of BMI on a group with only osteoporosis.

In comparing our results on general bone fractures in early BC with those in the literature, we found that in the EBCTCG trial, a meta-analysis of individual patient data comprising 18,766 women with early BC from 26 randomized clinical trials, bone fracture rates in post-menopausal women were 10.4% with AI therapy (five years) and 7.1% (five years) in those undergoing TAM [16]. The rates of bone fractures in the placebo-controlled arm of ABSCG-18 and AZURE trials were 9.6% and 8.3%, respectively, lower than in our study, which is hardly surprising given that these were randomized clinical trials involving a selected pre- and post-menopausal BC population treated with adjuvant BMAs versus placebo [7,31]. We, on the other hand, focused on a real-life population evaluated as part of a bone health management program in an Osteoncology Center. In the pre-menopausal setting, the combined analysis of the TEXT and SOFT trials on early-stage BC patients undergoing ovarian suppression in combination with TAM or AIs showed similar rates of bone fractures to those of our study (5.2% and 6.8%, respectively) [32].

Clinical outcome results in post-menopausal BC patients did not reveal a significant difference in DRFS between patients previously treated with BMAs and those had not received these agents. Our study also has limitations such as its retrospective nature and the involvement of a heterogeneous population of BC patients in terms of tumor stage, chemotherapy and hormone treatments administered, and duration of ET. In addition, some patients performed a QUS rather than a DXA analysis. QUS and DXA scans measure different bone characteristics, (bone quality and bone quantity, respectively) and therefore both can provide useful integrative information. However, several studies performed in large groups of healthy subjects have shown that QUS is as effective as DXA in predicting the risk of fracture [33,34]. The information collected on lifestyle was self-reported by patients and their answers may have been affected by response bias, i.e., an individual’s tendency to respond in a certain way regardless of the question. The overall low number of events may have confounded results, especially with regard to clinical outcome in both pre- and post-menopausal populations. Another issue is the limited median observation time in a percentage of women with a follow-up of < 5 years. A longer follow-up could help to explain long-term clinical outcome in this early BC population. The lack of correlation between CTX and bone fractures may have been due to the small number of events observed.

Changes in bone biomarkers could be used to monitor the biochemical effects of AIs on bone metabolism in early BC and to correlate these with the risk of future fracture risk in specific groups. However, they are still not routinely used in clinical practice [35,36]. We only analyzed the first CTX measurement as our study focused on BC patients at their initial Osteoncology clinical evaluation. The clinical impact of bone biomarkers on skeletal fractures warrants further investigation, especially in a cancer population at high risk of CTIBL. Notably, our study also included patients with a previous history of osteoporosis or other risk factors for bone fractures, which is uncommon in research evaluating bone health management in a cancer population [8].

## 5. Conclusions

Bone health evaluation in the adjuvant setting of BC is a multifactorial process that takes into account several risk factors, including ET, BMD, and lifestyle. The present real-life experience described the clinical impact of a bone health program in an Osteoncology Center of an Italian cancer institute aimed at preventing bone fractures in a large early BC population undergoing ET. Our findings confirm the importance of bone health maintenance in cancer populations, which should be considered as an important component of long-term cancer care.

## Figures and Tables

**Table 1:** Patient characteristics (*n* = 1125).

Characteristics	Total *n* = 1125 (%)	Pre-Menopausal *n* = 209 (%)	Post-Menopausal *n* = 916 (%)
Median age, years (range)	61 (26–88)	46 (26–63)	64 (31–88)
Daily dietary calcium intake			
≥RDA	824	140 (67.0)	684 (74.7)
<RDA	301	69 (33.0)	232 (25.3)
Normal alcohol consumption			
Yes	198	26 (13.8)	172 (20.5)
No	828	162 (86.2)	666 (79.5)
*Missing*	*99*	*21*	*78*
Normal physical activity			
Yes	732	139 (67.5)	593 (65.2)
No	384	67 (32.5)	317 (34.8)
*Missing*	*9*	*3*	*6*
Smoking habits			
Current or former smoker	223	44 (21.3)	179 (19.7)
Never smoker	893	163 (78.7)	730 (80.3)
*Missing*	*9*	*2*	*7*
Back pain			
Yes	594	98 (47.3)	496 (55.2)
No	511	109 (52.7)	402 (44.8)
*Missing*	20	2	18
*Bisphosphonate therapy*			
Yes	156	12 (94.0)	144 (16.3)
No	932	190 (6.0)	742 (83.7)
*Missing*	*37*	*7*	*30*
*Body Mass Index (BMI)*			
<25	569	141 (67.8)	428 (47.0)
25–29	381	51 (24.5)	330 (36.3)
≥30	168	16 (7.7)	152 (16.7)
*Missing*	*7*	*1*	*6*
CTX < 0.6	447	84 (64.6)	363 (63.4)
CTX ≥ 0.6	256	46 (35.4)	210 (36.6)
*Missing*	*422*	*79*	*343*
BMD measurement			
Spine DXA scan	435	109 (56.5)	326 (38.0)
Hip DXA scan	272	3 (1.6)	269 (31.4)
Qus	339	79 (40.9)	260 (30.6)
X-rays			
Cervical	36	6 (2.9)	30 (3.3)
Lumbar	455	82 (39.2)	373 (40.7)
Dorsal	668	115 (54.9)	553 (60.4)
Not specified	6	6	-
Bone mineral density (BMD)			
T score ≤ −2.5	333	38 (19.7)	295 (34.4)
T score > −2.5 and ≤ −1.0	500	90 (46.6)	410 (47.8)
T score > −1.0	217	65 (33.7)	152 (17.7)
*Missing*	*75*	16	*59*
Endocrine therapy			
AI + AI + TAM	780	-	780 (85.2)
TAM	136	-	136 (14.8)
LHRH + AI and LHRH alone	87	87 (41.6)	-
Other	122	122 (58.4)	-
Vitamin D level (ng/mL)			
≤10	192	19 (9.5)	173 (19.9)
>10	875	180 (90.5)	695 (80.1)
PTH			
Normal	838	169 (92.3)	669 (85.1)
Abnormal	131	14 (7.7)	117 (14.9)
*Missing*	*156*	*26*	*130*
pT			
T0–T1	779	145 (75.5)	634 (75.5)
T2–T3	253	47 (24.5)	206 (24.5)
*Missing*	*96*	*17*	*76*
pN			
N0	648	113 (61.7)	535 (69.0)
N1	310	70 (38.3)	240 (31.0)
*Missing*	*165*	*26*	*139*

RDA: Recommended Daily Allowance; CTX: Carboxy-terminal telopeptide of type I collagen; AI: Aromatase inhibitor; TAM: Tamoxifen; LHRH: Luteinizing hormone-releasing hormone; pT: Primary tumor; pN: Regional lymph nodes; PTH: Parathyroid hormone.

**Table 2:** Univariate and multivariate risk of morphometric vertebral deformities in pre-menopausal women.

Characteristics	Total *n* = 209 (%)	Fracture *n* = 10 (%)	No Fracture *n* = 199 (%)	Univariate OR (95% CI)	Multivariate OR (95% CI)
Median age, years (range)	46 (26–72)	47.5 (44–55)	45 (26–63)	1.14 (1.01–1.29)	1.10 (0.97–1.26)
Dietary daily calcium intake					
≤RDA	140 (67.0)	10 (100.0)	130 (65.3)	–	-
<RDA	69 (33.0)	0 (0.0)	69 (34.7)		
Normal physical activity					
No	67 (32.5)	4 (40.0)	63 (32.1)	1.00	-
Yes	139 (67.5)	6 (60.0)	133 (67.9)	0.71 (0.19–2.60)	
Smoking habits					
Never smoker	163 (78.7)	9 (90.0)	154 (78.2)	1.00	-
Current or former smoker	44 (21.3)	1 (10.0)	43 (21.8)	0.40 (0.04–3.22)	
Back pain					
No	109 (52.7)	5 (50.0)	104 (52.8)	1.00	-
Yes	98 (47.3)	5 (50.0)	93 (47.2)	1.12 (0.31–3.99)	
Bisphosphonate therapy					
No	190 (94.1)	8 (80.0)	182 (94.8)	1.00	
Yes	12 (5.9)	2 (20.0)	10 (5.2)	4.55 (0.85–24.29)	-
Body Mass Index (BMI)					
<25	141 (67.8)	8 (80.0)	133 (67.2)	–	-
25–29	51 (24.5)	2 (20.0)	49 (24.7)		
≥30	16 (7.7)	0 (0.0)	16 (8.1)		
CTX					
CTX < 0.6	84 (64.6)	6 (75.0)	78 (63.9)	1.00	-
CTX ≥ 0.6	46(35.4)	2 (25.0)	44 (36.1)	0.59 (0.11–3.05)	
Bone mineral density (BMD)					
T score > −1.0	65 (37.2)	1 (10.0)	64 (35.0)	1.00	1.00
T score > −2.5 and ≤ −1.0	90 (43.1)	2 (20.0)	88 (48.1)	1.45 (0.12–16.38)	1.42 (0.12-16.11)
T score ≤−2.5	38 (19.7)	7 (70.0)	31 (16.9)	14.45 (1.70–122.67)	11.6 (1.34-101.1)
Endocrine therapy					
LHRH+AI or LHRH alone	87 (41.6)	1 (10.0)	86 (43.2)	1.00	-
LHRH+TAM or TAM+AI+LHRH	122 (58.4)	9 (90.0)	113 (56.8)	6.85 (0.85–55.09)	
Vitamin D level (ng/mL)					
>10	180 (90.5)	9 (90.0)	171 (90.5)	1.00	-
≤10	19 (9.5)	1 (10.0)	18 (9.5)	1.05 (0.12–8.81)	
PTH					
Normal	169 (92.3)	10 (100.0)	159 (91.1)	–	-
Abnormal	14 (7.7)	0 (0.0)	14 (8.1)		
pT					
T0–T1	145 (75.5)	7 (77.8)	138 (75.4)	1.00	-
T2–T3	47 (24.5)	2 (22.2)	45 (24.6)	0.87 (0.17–4.37)	
pN					
N0	113 (61.7)	6 (75.0)	107 (61.1)	1.00	
N1	70 (38.3)	2 (25.0)	68 (38.9)	0.52 (0.10–2.67)	

RDA: Recommended Daily Allowance; OR: Odds ratio; CTX: Carboxy-terminal telopeptide of type I collagen; AI: Aromatase inhibitor; TAM: Tamoxifen; LHRH: Luteinizing hormone-releasing hormone; pT: Primary tumor; pN: Regional lymph nodes; PTH: parathyroid hormone.

**Table 3:** Univariate and multivariate risk of morphometric vertebral deformities in post-menopausal women.

Characteristics	Total *n* = 916 (%)	Fracture *n* = 161 (%)	No Fracture *n* = 755 (%)	Univariate OR (95% CI)	Multivariate OR (95% CI)
Age, years (categorized)					
≤65	508 (55.5)	59 (36.6)	449 (59.5)	1.00	1.00
>65	408 (44.5)	102 (63.4)	306 (40.5)	2.53 (1.78–3.60)	2.16 (1.42–3.26)
Dietary daily calcium intake					
≥RDA	684 (74.6)	130 (80.7)	554 (73.4)	1.52 (0.99–2.32)	–
<RDA	232 (25.4)	31 (19.3)	201 (26.6)	1.00	
Physical activity					
No	317 (34.8)	69 (43.1)	248 (33.1)	1.00	1.00
Yes	593 (65.2)	91 (56.9)	502 (66.9)	0.65 (0.46–0.92)	0.70 (0.47–1.05)
Smoking habits					
Current or former smoker	179 (19.7)	28 (17.5)	151 (20.2)	0.84 (0.53–1.31)	–
Never smoker	730 (80.3)	132 (82.5)	598 (79.8)	1.00	
Back pain					
No	402 (44.8)	55 (34.8)	347 (46.9)	1.00	1.00
Yes	496 (55.2)	103 (65.2)	393 (53.1)	1.65 (1.16–2.36)	1.54 (1.02–2.30)
Bisphosphonate therapy					
No	742 (83.7)	120 (77.4)	622 (85.1)	1.00	–
Yes	144 (16.3)	35 (22.6)	109 (14.9)	1.66 (1.08–2.55)	
Body Mass Index (BMI)					
BMI < 25	428 (47.0)	56 (36.2)	372 (49.5)	1.00	1.00
BMI 25–29	330 (36.3)	71 (44.7)	259 (34.5)	1.82 (1.23–2.67)	1.44 (0.92–2.27)
BMI ≥ 30	152 (16.7)	32 (20.1)	120 (16.0)	1.77 (1.09–2.86)	1.31 (0.73–2.33)
CTX					
CTX < 0.6	363 (63.4)	61 (59.2)	302 (64.3)	1.00	–
CTX ≥ 0.6	210 (36.6)	42 (40.8)	168 (35.7)	1.23 (0.80–1.91)	
Bone mineral density (BMD)					
T score > −1.0	152 (17.7)	20 (13.1)	132 (18.8)	1.00	1.00
T score > −2.5 and ≤ −1.0	410 (47.8)	62 (40.5)	348 (49.4)	1.17 (0.68–2.02)	1.10 (0.61–1.78)
T score ≤ −2.5	295 (34.4)	71 (46.4)	224 (31.8)	2.09 (1.21–3.59)	1.97 (1.07–3.60)
Endocrine therapy					
AI or TAM+AI	780 (85.2)	153 (95.0)	627 (83.0)	1.00	1.00
TAM + other	136 (14.8)	8 (5.0)	128 (17.0)	0.25 (0.12–0.53)	0.37 (0.15–0.89)
Vitamin D level (ng/mL)	-				
>10	695 (80.1)	115 (73.7)	580 (81.5)	1.00	1.00
≤10	173 (19.9)	41 (26.3)	132 (18.5)	1.57 (1.05–2.34)	1.41 (0.90–2.22)
PTH					
Normal	669 (85.1)	119 (87.5)	550 (84.6)	1.00	–
Abnormal	117 (14.9)	17 (12.5)	100 (15.4)	0.79 (0.45–1.36)	
pT					
T0–T1	634 (75.5)	102 (68.0)	532 (77.1)	1.00	1.00
T2–T3	206 (24.5)	48 (32.0)	158 (22.9)	1.58 (1.07–2.33)	1.26 (0.81–1.96)
pN					
N0	535 (69.0)	94 (68.1)	441 (69.2)	1.00	–
N1	240 (31.0)	44 (31.9)	196 (30.8)	1.05 (0.71–1.56)	

RDA: Recommended Daily Allowance; OR: Odds ratio; CTX: Carboxy-terminal telopeptide of type I collagen; AI: Aromatase inhibitor; TAM: Tamoxifen; LHRH: Luteinizing hormone-releasing hormone; pT: Primary tumor; pN: Regional lymph nodes; PTH: Parathyroid hormone.

## Data Availability

The datasets generated and/or analysed during the current study are available from the corresponding author on reasonable request.
